# Cardiovascular magnetic resonance in systemic hypertension

**DOI:** 10.1186/1532-429X-14-28

**Published:** 2012-06-11

**Authors:** Alicia M Maceira, Raad H Mohiaddin

**Affiliations:** 1 Cardiac Imaging Unit, ERESA Clinic, C/ Marqués de San Juan, 6, 46015, Valencia, Spain; 2 Royal Brompton and Harefield NHS Foundation Trust, London, UK; 3 National Heart and Lung Institute, Imperial College London, London, UK

## Abstract

Systemic hypertension is a highly prevalent potentially modifiable cardiovascular risk factor. Imaging plays an important role in the diagnosis of underlying causes for hypertension, in assessing cardiovascular complications of hypertension, and in understanding the pathophysiology of the disease process. Cardiovascular magnetic resonance (CMR) provides accurate and reproducible measures of ventricular volumes, mass, function and haemodynamics as well as uniquely allowing tissue characterization of diffuse and focal fibrosis. In addition, CMR is well suited for exclusion of common secondary causes for hypertension. We review the current and emerging clinical and research applications of CMR in hypertension.

## Review

Systemic hypertension is a highly prevalent [1] chronic disease, known to be a significant cardiovascular risk factor [2,3]. Longstanding hypertension causes significant damage to several organs such as the brain, eye, kidney and heart. Features of hypertensive heart disease include left ventricular hypertrophy (LVH), diastolic and eventually systolic heart failure.

A number of imaging techniques are available in the assessment of hypertension, including echocardiography, cardiovascular magnetic resonance (CMR), cardiac computed tomography (CCT) and cardiac scintigraphy. 2D echocardiography is widely available and extensive literature supports its indication for left ventricular (LV) mass measurement. This technique can quantify global and regional systolic function, as well as diastolic function. 3D echocardiography has partially overcome problems with accuracy and reproducibility, while difficulties due to poor acoustic windows and attenuation still persist. CCT has a high negative predictive value for ruling out coronary artery disease and it is also appropriate for ventricular mass and volume measurement and for assessing vascular disease, while more extensive research is needed before its use for myocardial tissue characterization can be promoted. Modern CT scanners generate significantly lower doses of ionizing radiation, but these are not widely available and issues with the use of nephrotoxic contrast still remain. Equally, scintigraphic techniques can be used in the assessment of hypertensive heart disease but their use is greatly limited by the limited spatial resolution and thus limited accuracy in determining LV mass. Thus, CMR appears as the one technique to provide a comprehensive assessment of hypertensive cardiovascular disease, including accurate and reproducible measurement of biventricular function and volumes, the possibility of serial evaluation, assessment of valvular disease and vascular pathology, myocardial perfusion and tissue characterization.

Cardiac specific sequences are usually implemented in 1.5 T scanners, though 3 T and 1 T open scanners can be also used. They all yield good image quality with high spatial resolution. Steady state free precession (SSFP) cines, phase contrast sequences, T2-weighted short-tau inversion recovery (STIR), T1- and T2-weighted fast spin-echo, perfusion and myocardial late gadolinium enhancement (LGE) sequences along with 3D-magnetic resonance angiography (MRA), are the most widely used sequences in hypertension. Other CMR techniques such as spectroscopy, myocardial tagging, T1 relaxometry-based techniques and myocardial velocity mapping could be useful in the future though at present they are limited to the research arena. The utility and advantages of CMR in the characterization of hypertensive cardiac and vascular disease, as well as in detection of secondary hypertension, are reviewed below.

### Pathophysiological consequences of hypertension in the cardiovascular system

Longstanding hypertension has deleterious effects on several target organs, including heart, brain, kidneys, eyes and vessels. Regarding the cardiovascular system, hypertensive heart diseases includes left ventricular hypertrophy, myocardial fibrosis, diastolic prior to systolic ventricular dysfunction and heart failure, atrial myopathy, atrial fibrillation, microangiopathy with decreased coronary reserve, and development of epicardial coronary stenoses [4]. With regard to the vascular system, the main findings are endothelial dysfunction, remodelling of the small and large arteries with reduced dilatation capability, and generalized atherosclerotic changes including the development of stenoses and aneurysms.

CMR has a unique potential to visualize and assess all of these alterations in hypertensive patients, leading to an accurate diagnosis.

### Indications and protocol for CMR in hypertension

According to the Joint National Committee VII report on the prevention, detection, evaluation and therapy of hypertension (JNC-VII) [5], two of the objectives in the management of hypertensive patients are to assess target organ damage and to detect identifiable causes of hypertension. In both, CMR can be useful though no specific indications for this imaging technique in hypertensive heart disease have been established so far and no information regarding CMR is provided in the above mentioned report.

The 2007 ESH/ESC guidelines [6] recommend total cardiovascular risk be evaluated in each patient to decide about important aspects of treatment such as the threshold at which to commence drug administration, the target blood pressure to be reached, the use of drug combinations as the initial treatment step, and the possible addition of other agents to treatment. According to these guidelines, the recommended imaging technique when a more sensitive detection of left ventricular hypertrophy than that provided by ECG is needed is echocardiography, particularly in patients in whom organ damage is not detected by ECG and in the elderly in whom cardiac hypertrophy is frequent. These guidelines indicate that other imaging techniques such as CMR should be reserved for specific indications which are unfortunately not summarised in the text. Also, as discussed in greater detail below, 3D-gadolinium enhanced MR angiography is considered the imaging technique of choice for investigating renovascular hypertension, while MR imaging is also considered a good option to localize phaeochromocytoma and primary aldosteronism once an endocrine diagnosis has been made. In the 2009 Reappraisal of European guidelines on hypertension management [7], the predictive value of both ECG and echocardiography were acknowledged with new additional evidence provided, while no specific recommendation was done on the use of CMR. Finally, in the 2010 ACCF/ACR/AHA/NASCI/SCMR Expert Consensus Document [8] no information regarding the usefulness of CMR in hypertension was included.

**Figure 1:**
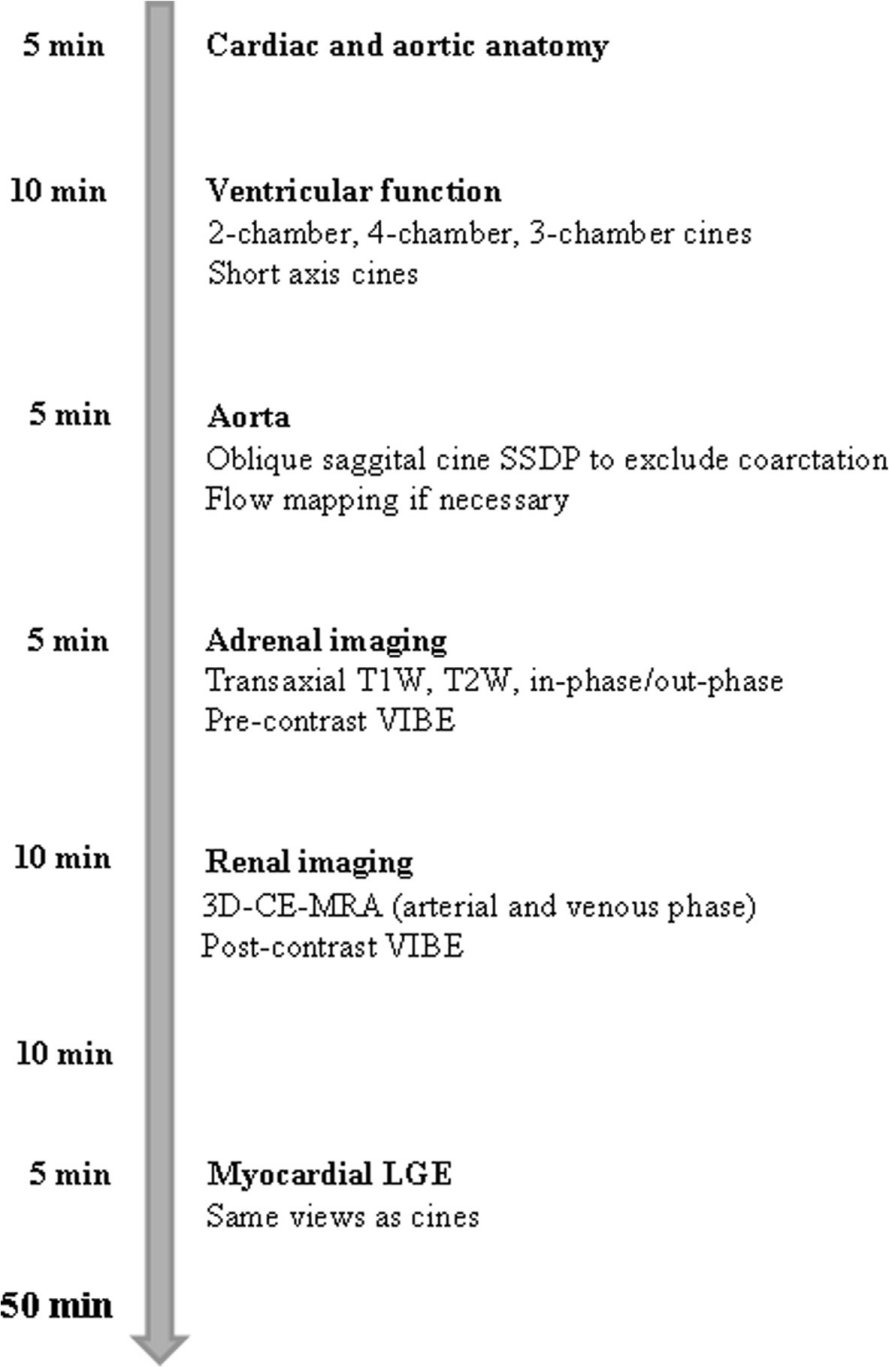
Time-line figure for the proposed standard CMR protocol in hypertension.

A proposed standard CMR protocol in hypertension is depicted in Figure 1. In brief, the protocol includes a transaxial set of SSFP or black blood fast spin echo images through the chest. If necessary also coronal and sagittal orientations can be acquired. Next, high spatial resolution SSFP cine sequences are taken in three long axis views (2 chamber, 4 chamber and LV outflow tract) and in a short axis stack from the atrioventricular valves through the apex. There images are eventually used for ventricular volume and mass measurements. An SSFP cine in oblique sagittal of the aorta should also be acquired to rule out aortic coarctation. If any sign of coarctation is seen, black blood high resolution turbo spin echo (TSE) sequences should be acquired, as well as velocity-encoded cines at the level of the coarctation and at the diaphragmatic level followed by 3D-MRA. Thereafter, T1W, T2W, in-phase and out-of-phase pre-contrast VIBE adrenal imaging and 3D-MRA of the renal arteries is performed. Finally, 10 minutes after this last acquisition, late gadolinium enhancement (LGE) myocardial imaging should be acquired in the same views as the previously acquired cine sequences.

### Value of CMR in the assessment of hypertensive cardiovascular disease

Both the heart and the aorta are target organs of systemic hypertension. Hypertensive heart disease is characterized by LVH, left atrial dilatation, myocardial fibrosis, diastolic prior to systolic dysfunction, and increased incidence of coronary artery disease. Hypertensive vascular disease manifests as aortic wall pathology such as increased prevalence of atheromatous plaques and higher risk of aneurysm, dissection, penetrating ulcer, intramural hematoma or peripheral obstructive disease.

### Measurement of ventricular dimensions, geometry and systolic function

The Framingham Study showed that increases in LV mass are associated with significant increments in cardiovascular morbidity and mortality independent of the presence of coronary artery disease, systemic hypertension or other cardiovascular risk factors [2,9], with a three-fold increase in mortality both in patients with [2,10] and without [11] these conditions. For these reasons, the JNC VII report [5] recommends more intensive management in patients with LVH.

Electrocardiographic criteria of LVH have been in use for several decades [12,13] but these criteria were mostly validated against M-mode echocardiography [14] and lacked sensitivity for LVH. They have been recently recalibrated against CMR [15], with improved diagnostic accuracy. At present, imaging techniques such as echocardiography and CMR are preferred for detecting LVH.

Implementation of echocardiography in its M-mode and especially 2D modalities dramatically changed cardiac assessment. This technique has great advantages such as availability, safety and price, for which it is considered the first line imaging technique in the assessment of heart dimensions and function. Nonetheless, it has several significant limitations such as its dependency on observer, patient anatomy and position, problems with acoustic window and with attenuation. M-mode echocardiography has been widely used in the past but, due to the need for geometric assumptions, its accuracy and reproducibility are very low [16]. More recently, accurate and reproducible 2D echocardiography techniques to measure volumes and mass based on Simpson’s rule have attained widespread use, though they still entail geometric assumptions that cause inaccuracy especially in dilated, deformed ventricles [17]. The advent of three-dimensional echocardiography, which does not require geometric assumptions, has produced measurements comparable to CMR [18], with good accuracy and reproducibility. However, numerous limitations remain such as poor acoustic windows and attenuation, as well as the need for experienced observers.

#### Measurement of ventricular volumes by CMR

CMR is currently considered the gold standard for the measurement of right and left ventricular volumes, mass and function, boasting high accuracy and reproducibility [17,19]. Reference values have been published for all these parameters [20]. With modern SSFP cine sequences combined with parallel processing techniques, a whole contiguous ventricular short axis cine stack can be acquired in potentially just one breath-hold. In one study, in addition to accurate assessments of ventricular volumes and function, an overall assessment of cardiac and extracardiac (thoracic) anatomy can be obtained. Published data reveal an intraobserver and interobserver variability of 2.0–7.4% and 3.3–7.7%, respectively [19]. These values are lower than those obtained with echocardiography, conferring lower sample size requirements for intervention studies in several conditions such as heart failure or hypertension and thereby also dramatically reducing study costs [21].

Importantly, recent data have shown that LV mass should be indexed to height to the power of 1.7, which is more sensitive than body surface area indexed LV-mass in identifying obesity-related LVH, and appears to be most consistently associated with cardiovascular events and all-cause mortality [22].

#### LV geometry

CMR can not only quantify LV mass but also assess the pattern of hypertrophy, that is, LV geometry, which may have prognostic importance. The assessment of LV remodelling by echocardiography has the same limitations as related above. By contrast, the unfettered full myocardial coverage of CMR offers a precise measurement of LV wall thickness [23] and geometry and has provided other measures of LV remodelling and hypertrophy including the relative wall mass, which is the conceptual equivalent of the echocardiogram-derived relative wall thickness [24,25]. The relative wall mass is calculated by dividing the LV mass by the LV end-diastolic volume, and effectively indexes ventricular wall thickness to cavity size. Thus, concentric or eccentric LV hypertrophy (increased LV mass with relative wall mass above or below the cut-off value of 1.16, respectively) and concentric remodelling (normal LV mass with relative wall mass above 1.16) all predict an increased incidence of cardiovascular disease, but concentric hypertrophy has consistently been shown to be the condition which most markedly increases risk [26]. In addition, differences in the prevalence of LVH and different ventricular remodelling patterns have been identified in various ethnic groups and should be taken into account when evaluating cardiovascular risk in these populations [27].

#### CMR and LV function

Ejection fraction as well as endocardial and mid-wall fractional shortening have all been proposed as possible additional predictors of cardiovascular events in hypertension [28], though CMR has recently shown that midwall fractional shortening has limitations related to appropriateness for assessment of cardiac pumping, and dependency of LV wall thickness [29]. While conventional high temporal resolution cine sequences allow for an accurate assessment of the overall ventricular performance, if the aim is to study regional function, deformation or strain measured by CMR tissue tagging is indicated [30]. A number of recent studies have shown that though global ejection fraction is preserved in the early stages of hypertension, mid-wall shortening measured by echocardiography and circumferential strain measured by CMR tagging are depressed, with a reduction that appears to be associated with raised blood pressure [31], and, furthermore, a substantial regional intramyocardial strain heterogeneity can be observed, with most severely depressed strain patterns in the septum [32]. Myocardial tagging could thus be applied to characterize the physiological contraction cycle, to determine pathophysiological changes of rotation, longitudinal and circumferential shortening, and to follow-up changes of LV wall motion in hypertensive LVH.

### CMR in hypertrophy regression studies

**Figure 2:**
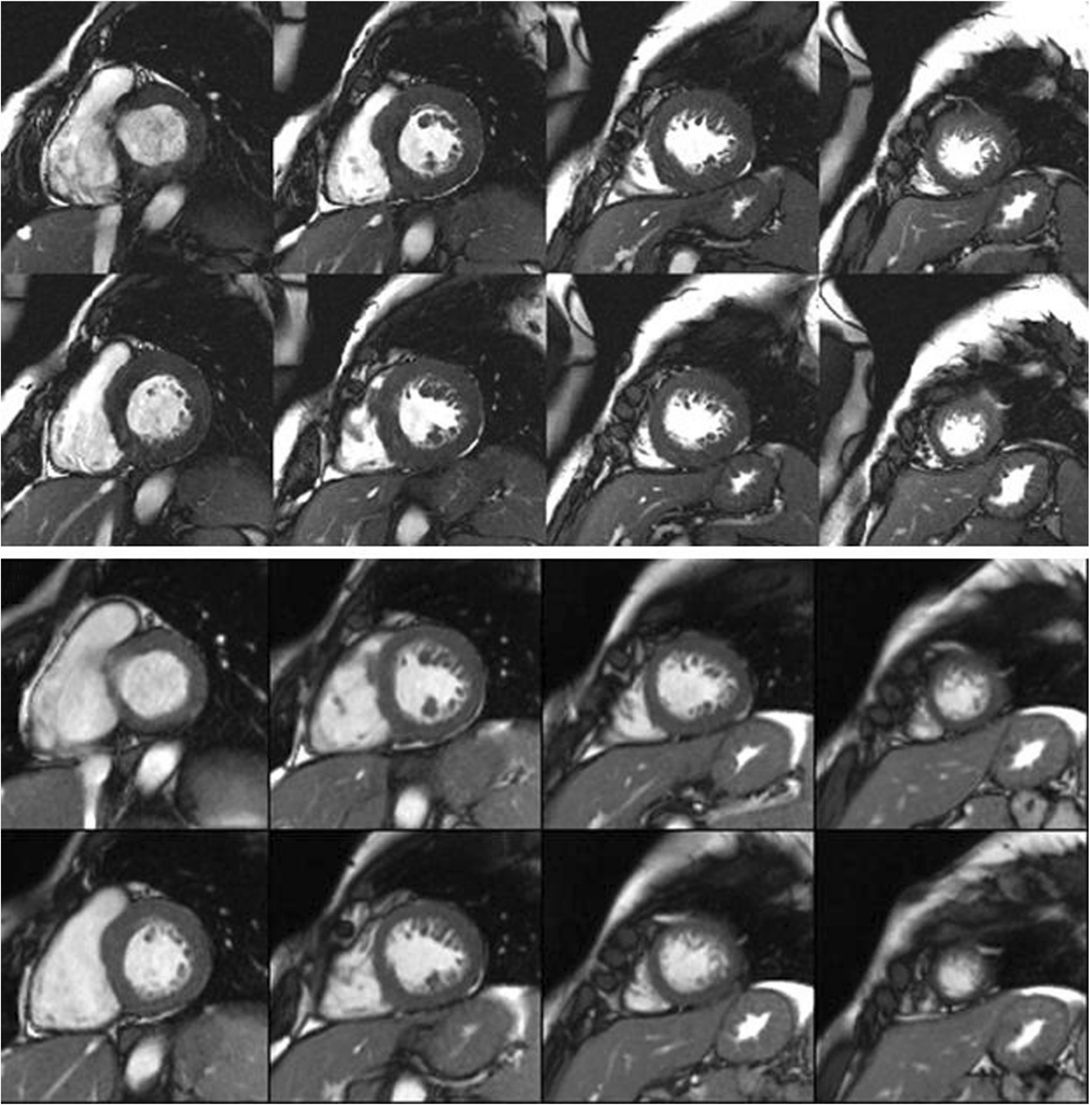
**Regression of hypertrophy after excision of an adrenal adenoma.** Panel A shows a baseline short axis stack of a 41 year old female patient with resistant hypertension, LV mass = 232 g. Panel B shows the same patient 1 year after excision of an adrenal adenoma, LV mass = 154 g.

While LVH of whatever aetiology confers increased cardiovascular risk, regression of LVH has been shown to be associated with decreased risk [33]. Due to its high reproducibility, CMR has been used for measurement of ventricular mass regression in a number of intervention studies in hypertension. These include a LIFE substudy in which contribution of high blood pressure to ventricular remodelling was assessed [34]; the TELMAR study [35], which compared the effect of telmisartan to metoprolol on LVH in patients with uncontrolled hypertension; the LVH-4E [36], in which eplerenone was compared to enalapril or a combination of both in LVH regression in hypertensive patients; the ALIVE study [37], in which benazepril with either amlodipine or a diuretic was tested; and recently the ALLAY trial [38], in which aliskiren, a direct renin inhibitor, was shown to be as effective as losartan in reducing LV mass. Figure 2 illustrates a case of hypertrophy regression in a 41 yo woman with resistant hypertension, 1 year after excision of an adrenal adenoma.

### Differential diagnosis with other causes of LVH

Determination of the aetiology of LVH is a common clinical problem that can be challenging, because pathological forms of LVH present with overlapping cardiac hypertrophy phenotypes. A number of other conditions can mimic LVH such as infiltrative diseases, hypertrophic cardiomyopathy (HCM), Fabry’s disease, cardiac sarcoidosis, aortic stenosis, and exercise-induced ventricular hypertrophy or athlete’s heart. While aortic stenosis is a straightforward differential diagnosis, other conditions can be difficult. The unique capacity of CMR to non-invasively characterise myocardial tissue represents an important advantage over other non-invasive imaging modalities. Regarding infiltrative conditions, a specific pattern of late gadolinium enhancement has been described for amyloid heart disease [39,40] with good sensitivity of 80%, specificity of 94% and positive predictive value of 92% that has simplified the differential diagnosis.

In a recently published study multiparametric CMR imaging was used to differentiate hypertensive LVH from HCM and distinctive hypertrophic phenotypes were detectable [41]. While hypertensive patients had reduced ejection fraction, increased cardiac chamber volumes, increased LV wall stress and reduced anteroseptal systolic strains, patients with HCM had supernormal ejection fraction, reduced LV wall stress, decreased longitudinal systolic strain and fibrosis. Logistic regression analysis identified increased LV wall stress as the hallmark of hypertension (odds ratio 1.2, SE 0.01, specificity 80%, sensitivity 65.5%, diagnostic accuracy 72.9%), while HCM was best characterized by reduced total longitudinal strain (odds ratio 1.3, SE 0.02, specificity 89.3%, sensitivity 78.6%, diagnostic accuracy 78.6%).

**Figure 3:**
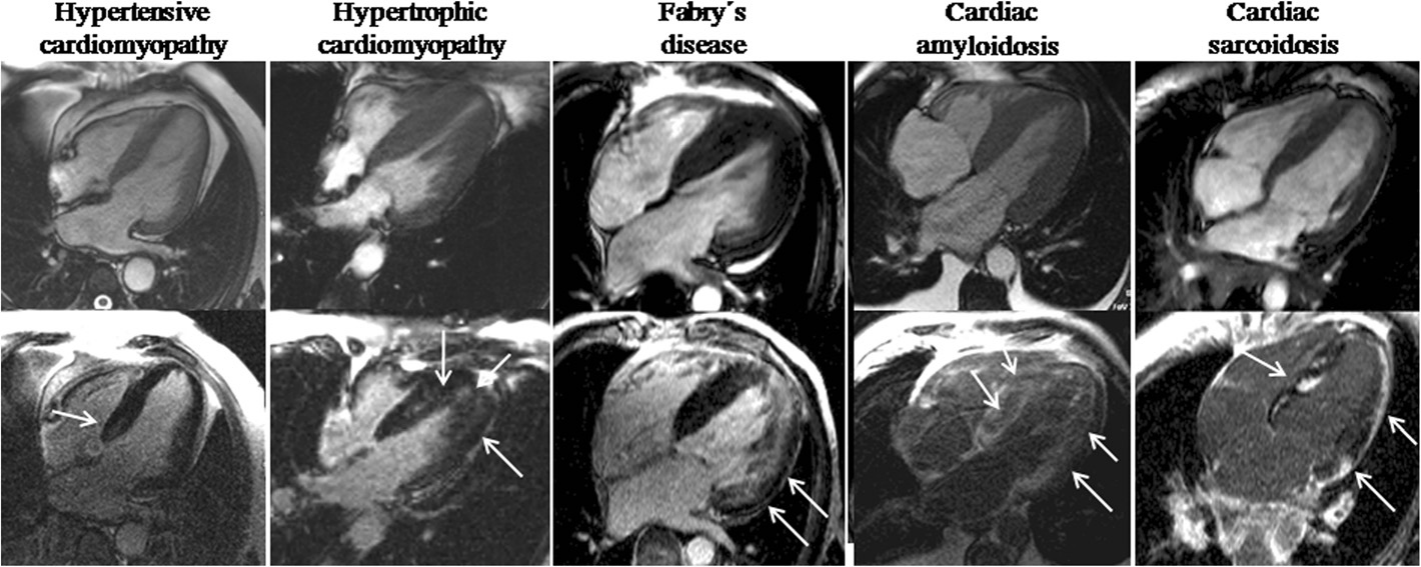
**Differential diagnosis of LVH.** Diastolic SSFP cines (above) and LGE-sequences (below) in the 4-chamber view of several types of hypertrophy are depicted. From left to right: hypertensive cardiomyopathy, hypertrophic cardiomyopathy, Fabry’s disease, cardiac amyloid infiltration and cardiac sarcoidosis. Different gadolinium myocardial enhancement patterns (arrows) are shown corresponding to each condition: very subtle focal intramyocardial fibrosis in the basal septum of a hypertensive cardiomyopathy patient, confluent intramyocardial fibrosis in a case of hypertrophic cardiomyopathy, subendocardial fibrosis in the lateral wall in a case of Fabry’s disease, global difuse subendocardial fibrosis in amyloid cardiomyopathy and subendocardial/intramyocardial/subepicardial fibrosis in a case of sarcoidois.

Also, athlete’s heart can be distinguished from other forms of pathological hypertrophy with the CMR-derived diastolic wall-to-volume ratio [42]. A cut-off value of less than 0.15 mm × m^2^ /ml, is said to have a 99% specificity for sport-related LVH. However this finding requires further validation and confirmation in larger cohorts. Figure 3 depicts the differential diagnosis of LV hypertrophy.

### CMR and detection of myocardial fibrosis

Animal and human studies have shown that in hypertensive LVH accumulation of type I and III collagen fibres occurs both in the interventricular septum and free wall [43]. This increase in collagen content appears to be a frequent feature of LVH, regardless of its cause and just depending on the severity of LV remodeling [44]. Myocardial fibrosis can be focal, also called reparative or replacement fibrosis [45], or diffuse, also known as interstitial fibrosis and is the most typical pattern in hypertensive heart disease. Fibrosis would cause myocardial stiffness and subsequent changes in ventricular function, electrical activity and myocardial perfusion that may potentially affect prognosis [46].

Interest has recently grown in the possibility of assessing the degree of myocardial fibrosis in order to refine the ability to predict outcome in hypertensive LVH, and several methods have been proposed. Serum markers of fibrosis have been proposed, mainly the carboxyterminal propeptide of procollagen type I (PIP), a marker of collagen synthesis whose serum levels correlate with the presence of severe myocardial fibrosis [47]. Collagen markers however lack specificity as they are only partly derived from the heart [48]. Among the imaging techniques, a simple Doppler-derived index such as the mitral inflow deceleration time has been used to calculate myocardial stiffness as an indirect marker of myocardial fibrosis [49]. Echocardiographic analysis of cyclic variation of the backscatter signal has also been proposed used for this purpose and shown to vary according to serum PIP levels [50] and diastolic function [51]. However, echocardiography only provides an indirect assessment of fibrosis, and is plagued with reproducibility problems, attenuation, artefacts and difficulty in achieving correct alignment of the beam, especially for Doppler-based techniques. Speckle tracking has partially overcome these limitations as it does not depend on the correct alignment of the ultrasound beam, but it still requires high quality echocardiographic images for adequate endocardial border delineation.

**Figure 4:**
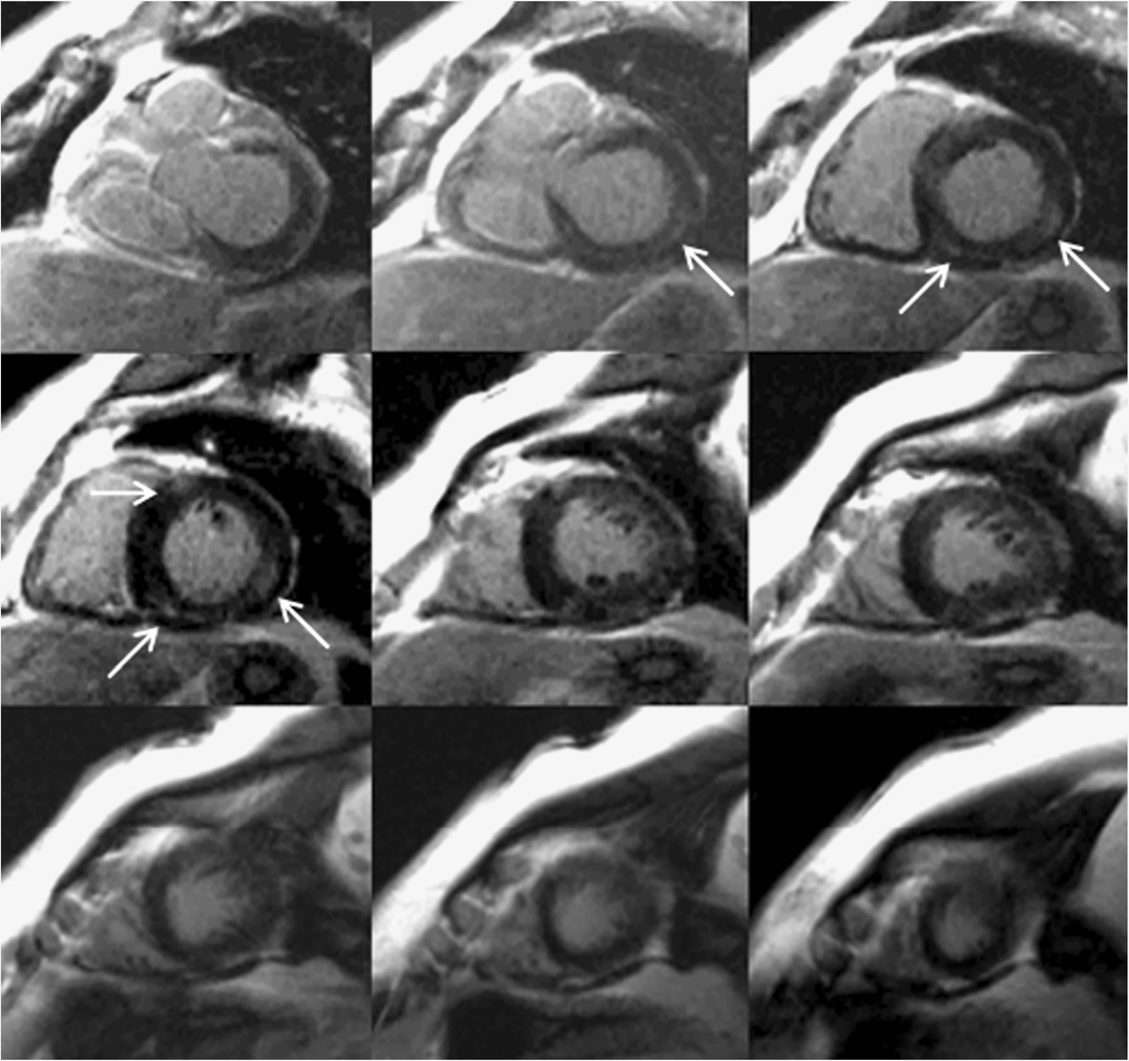
**Focal myocardial fibrosis in a hypertensive patient.** LGE-sequences in a 63 year old female patient with longstanding hypertension are shown. The arrows show an area of intramyocardial late gadolinium enhancement in the basal and mid inferoseptal and inferolateral segments. This is not the most typical fibrosis pattern in hypertension, which is usually diffuse.

LGE-CMR is able to show focal myocardial fibrosis, as demonstrated in several conditions including hypertension [39,44,52-54], a case of which is shown in Figure 4. In this respect one study has shown that 45% of patients with arterial hypertension had myocardial LGE, which appears to be related to both myocardial interstitial fibrosis and coronary microangiopathy [55]. The main limiting factor of using LGE-CMR in hypertensive patients, though, is that the fibrotic process is often diffuse. Diffuse myocardial fibrosis is missed by LGE-CMR because LGE relies on relative differences in signal intensities and employs the myocardium with lowest signal intensity as a reference for normality, regardless of the degree of fibrosis contained in it. In this regard, the T1 mapping techniques have an advantage over LGE-CMR as they are based on images produced with a standardized scale calculating the relaxation time of each pixel within a parametric image. Each tissue exhibits a characteristic T1 relaxation time at a selected magnetic field strength that depends on tissue composition and, thus, deviation from normal ranges could be used to detect and quantify pathological processes such as hypertensive difuse myocardial fibrosis. Early T1 mapping techniques were very time consuming. A Look-Locker sequence [39,56-58] consisting of a gradient echo cine sequence with a nonslice selective inversion pulse after an R wave followed by a segmented gradient echo acquisition. This was applied both before and at least two times, from five minutes after contrast administration. Mean signal intensities were plotted against the delay after the inversion pulse. The points were then fit with a nonlinear least squares procedure and T1 values were derived from the fit parameters. Finally, a myocardial gadolinium contrast partition coefficient would be calculated from the change of relaxation rate after contrast injection. More recently, the MOdified Look-Locker Inversion recovery pulse sequence (MOLLI) was developed that merged images from three consecutive inversion-recovery experiments into one data set, generating single-breath-hold single-slice T1 maps of the myocardium [59-61]. A Shortened MOdified Look-Locker Inversion recovery (ShMOLLI) sequence [62] has also been recently tested [63]. This uses sequential inversion recovery measurements within a single shortened breath-hold. These sequences hold promise for the detection of interstitial myocardial fibrosis in patients with hypertensive heart disease.

### CMR in the assessment of diastolic performance, atrial dimensions and function

In longstanding systemic hypertension, diastolic heart failure, characterized by heart failure symptoms, preserved ejection fraction and diastolic dysfunction, often develops and carries an ominous prognosis [64]. The technique of choice for noninvasive assessment of diastolic function is echocardiography, mainly flow Doppler and tissue Doppler imaging. The assessment of diastolic function with CMR requires the use of high temporal resolution sequences. Several techniques have been used for this purpose, such as quantification of ventricular volume change over time in the cardiac cycle using retrospective gating, which provides parameters such as: atrial filling ratios, peak diastolic filling rate and time to peak filling rate; analysis of three dimensional myocardial strains with tissue tagging gated to diastole, and mitral inflow velocity curves obtained with phase contrast sequences. A novel CMR-derived index - diastolic volume recovery, calculated as the percentage proportion of diastole required for recovery of 80% stroke volume, has been recently shown to yield the best performance versus echocardiography to detect diastolic dysfunction [65]. Still, the superior temporal resolution and ease of assessment of echocardiography in its multiple variants renders it by far the technique of choice for diastolic function assessment.

Atrial dilatation correlates with the chronicity of hypertension and with the severity of diastolic dysfunction. Importantly, atrial size is independently associated with cardiovascular morbidity and mortality [66] and with the development of atrial fibrillation [67]. While echocardiography calculates volumes using geometric assumptions, which due to the shape of the atria are prone to be highly inaccurate, CMR provides an accurate and reproducible measure of atrial volume by means of a 3D approach, in all types of patients [68]. Reference values of left atrial dimensions and volume with CMR are now available [69].

### CMR for the detection of coronary artery disease

Assessing coronary disease in the hypertensive patient with chest pain is particularly challenging. Systemic hypertension is an established risk factor for epicardial coronary artery disease [70,71], but, in the setting of hypertensive LVH, small vessel disease with impaired myocardial perfusion reserve can be responsible for myocardial ischemia without significant coronary artery stenosis by coronary angiography [72]. It has been suggested that stress perfusion CMR may non-invasively differentiate patients with small vessel disease from those with epicardial coronary artery disease, based on the temporal and spatial extent of the perfusion deficits. In small vessel disease perfusion deficits are usually diffuse, circumferential, affecting ≤ 1/3 of the wall thickness and with shorter persistence (≤ 5 heartbeats) than those in patients with significant epicardial coronary disease [73-75]. This differentiation is important with respect to therapy and also to prognosis [76].

Saturation-recovery sequences acquired on first pass of gadolinium during vasodilator stress with either adenosine or dipyridamole have a good diagnostic yield for detection of ischaemia and significant epicardial coronary disease [77-79], while late gadolinium enhancement CMR is considered the best technique for localization and quantification of myocardial necrosis [80,81].

### CMR in the assessment of aortic and peripheral vascular disease

**Figure 5:**
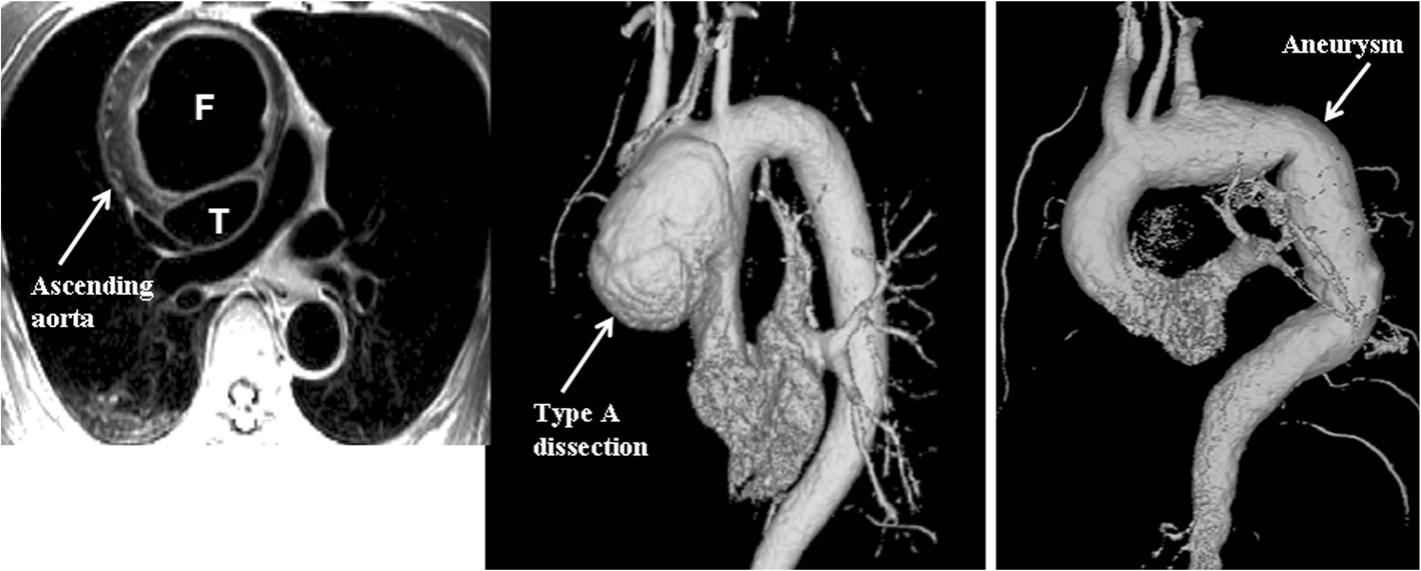
**Aortic wall pathology.** The left and middle pictures show a T1WTSE and 3D-MRA sequences of a type A aortic dissection, in which the true (T) and false (F) lumens can be clearly seen. The right picture shows an aortic aneurysm involving the arch and descending thoracic aorta of a hypertensive patient.

Systemic hypertension is an established risk factor for aortic and peripheral vascular disease. On the other hand, the relationship between cardiovascular risk factors including hypertension [82,83] and visualization of atheroma plaques in the aorta, as well as their prognostic value [84], has been reported. CMR can provide a comprehensive assessment of aortic anatomy and function, including not only the assessment and follow-up of aortic wall syndromes such as aneurysm, dissection or intramural hematoma, but also evaluation and quantification of the atherosclerotic plaque volume, composition, biological activity, and quantification of aortic functional parameters such as stiffness, distensibility and pulse wave velocity. Figure 5 illustrates the utility of T1WTSE and 3D MRA sequences in the assessment of a type A aortic dissection and aortic aneurysm.

Plaque imaging may be important as part of risk assessment, which may alter thresholds for treatment, and may be of utility in plaque-regression studies. It has been shown that patients with prior major cardiovascular events have greater plaque burden and plaque eccentricity [85]. Likewise, the presence of aortic atheroma in a particular patient indicates a higher risk of a cardiovascular event than could be expected from the Framingham score alone, which was not designed to detect atherosclerotic plaques [86]. Thus, reasonably, assessment of sub-clinical atherosclerosis could be considered in persons at intermediate risk by the Framingham score, in whom the presence of aortic atheroma might cause a change in therapy.

**Figure 6:**
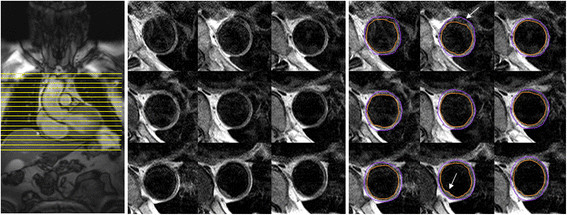
**Aortic wall volume measurement.** The middle picture show T2W-TSE continuous transverse slices of the thoracic aorta as piloted from the left picture. The right picture shows the same sequence with regions of interest for volumetric quantification. Arrows indicate the location of small aortic plaques.

Black blood CMR imaging is highly accurate and reproducible for assessment and volumetric quantification of atheroma plaques in the carotid arteries [87] and aorta [88,89], as illustrated in Figure 6. Recent MR technological improvements allow fast, accurate, and reproducible plaque imaging. Interestingly, multicontrast CMR is able to assess plaque composition, and markers of plaque inflammation and neovascularisation, such as superparamagnetic phagocytosable nanoparticles [90], have been developed for *in vivo* plaque imaging. Protocols for assessing aortic wall anatomy and structure usually include high resolution T1 and T2-weighted TSE and STIR sequences and/or 3D angiography. Images obtained are eventually processed in order to quantify plaque volume and characterize plaque composition, which have been histologically validated [91,92].

A functional assessment of the aorta can be carried out with CMR by acquiring flow-sensitive and cine sequences. Blood flow affects arterial remodelling, and decreased arterial distensibility is an early manifestation of adverse structural and functional changes within the vessel wall [87]. Furthermore, atherosclerosis, as well as endothelial biology, depends on arterial wall shear stress. Flow-sensitive 4D-MRI, which allows the depiction of 3D morphology as well as the acquisition of time-resolved blood flow velocities in 3 directions, provides information regarding the regional differences in absolute wall shear stress and oscillatory shear index. This may help explain the physiopathology of atherosclerotic lesions, mainly how they develop and progress predominantly at specific locations in the aorta [93].

### CMR in the detection of Secondary causes of Hypertension

Resistant hypertension is defined as failure to achieve target blood pressure when a patient adheres to optimal doses of three or more antihypertensive drugs usually including a diuretic [5]. The prevalence of resistant hypertension may range from 5% in general practitioner clinics to more than 50% in specialist hypertension clinics [94]. Secondary causes of hypertension are common in patients with resistant hypertension and recognition of these identifiable causes is important in order to offer the patient the most adequate and effective management and thereby improve prognosis by preventing the chronic complications of hypertension. Accordingly, the JNC VII report [5] recommends more intensive management if clinical data, physical signs or routine laboratory tests suggest secondary hypertension.

**Figure 7:**
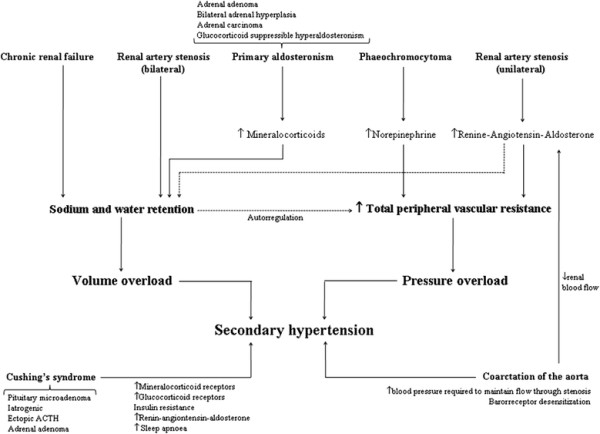
Pathophysiology of several secondary causes of hypertension: renovascular hypertension, primary aldosteronism, coarctation of the aorta, phaeochromocytoma and Cushing’s syndrome.

Common secondary causes are renal parenchymal disease, renal arterial disease, primary hyperaldosteronism and obstructive sleep apnoea. Less common causes are Cushing’s syndrome, phaeochromocytoma, thyroid disease, aortic coarctation, and intracranial tumours. The main physiopathological mechanisms are depicted in Figure 7. There are a number of imaging techniques, along with laboratory tests, that can be carried out to rule out secondary hypertension and some of them, summarized below, can be assessed in the course of a CMR study in a hypertensive patient in whom the clinical suspicion is high.

### Renovascular disease

This may be present in up to 35% of patients with resistant hypertension [95]. Of all cases of renal artery stenosis, 90% are of atherosclerotic origin [96] and 10% are due to fibromuscular dysplasia. The latter is considered a systemic disease in which renal arteries are the most frequent localization, though other arteries may also be involved. This condition develops mainly in women below 50 years of age, involves the mid and distal renal arteries and branchpoints, and treatment is angioplasty in most cases. On the other hand, atherosclerotic renal artery stenosis is usually found in patients with widespread atheromatous disease, clinical presentation includes severe hypertension, recurrent flash pulmonary oedema, progressive renal dysfunction and a higher incidence of adverse cardiovascular events [97]. Recommended management for atherosclerotic renal artery stenosis is medical therapy, while angioplasty has been traditionally reserved only to protect renal function but a recent study has shown substantial risks and no evidence of a worthwhile clinical benefit from revascularization [98]. The detection of bilateral renal artery stenosis can nevertheless guide pharmacotherapy.

**Figure 8:**
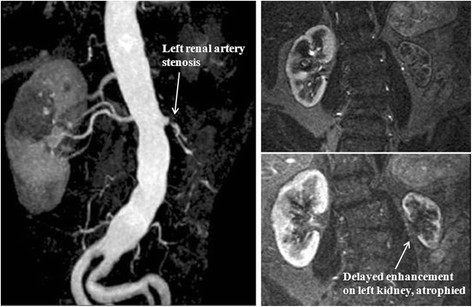
**3D-MRA of a 66 year old hypertensive patient with increased plasma creatinine on ACE-inhibitor therapy.** The study shows severe left renal artery stenosis. The raw images show delayed enhancement on the left kidney, which also shows atrophic changes.

Screening techniques for renal artery stenosis are warranted when there is a high level of suspicion (young women and older patients). There are a number of non-invasive tests available including duplex ultrasound, which provides anatomical information as well as flow velocity quantification and assessment of pressure wave morphology, CT-angiography, and nuclear captopril renal scintigraphy. The gold standard diagnostic test has traditionally been intra-arterial digital subtraction contrast angiography but this technique is invasive and not free of complications [94]. While for suspected fibromuscular dysplasia it may be reasonable to proceed directly to renal angiography, for most patients with suspected atherosclerotic disease this technique should be limited to those patients willing to undergo revascularization, the benefit of which is increasingly questioned. Magnetic resonance angiography (MRA) and functional renal MR imaging are promising alternatives that also allow for functional characterization of renal artery stenosis. MRA is highly sensitive for stenosis, but the specificity can be low and minimal lesions can be characterized as moderate or high grade [99]. Over the past few years, technical improvements including high-performance gradient systems, high parallel-imaging acceleration factors, and improved surface coil technology have allowed increased isotropic, sub-millimetre spatial resolution with high signal-to-noise ratios. By reducing scan and breath-hold time, these sequences have greatly improved the diagnosis of renal artery stenosis. Two meta-analyses [100,101] showed that gadolinium-enhanced 3D MRA has a sensitivity and specificity of >90%. Finally, the use of new higher-relaxivity blood-pool contrast agents for combined first-pass and steady-state imaging in patients with suspected renovascular hypertension has potential added value. Figure 8 illustrates the utility of 3D-MRA in a hypertensive patient with severe left renal artery stenosis in whom the raw images also show delayed enhancement and atrophic changes on the left kidney.

In the context of renal artery stenosis, which is usually associated with a degree of renal impairment, the issue of nephrogenic systemic fibrosis (NSF) must be considered prior to administration of gadolinium contrast. NSF is an extremely rare but important complication of gadolinium administration, associated with acute renal failure or severe chronic renal failure due to advanced (glomerular filtration rate less than 30 mL/min/1.73 m^2^) chronic kidney disease, but also with other conditions such as liver failure, liver or kidney transplant, hypercoagulability, deep vein thrombosis and proinflammatory processes. NSF is a scleroderma-like fibrosing entity which affects the skin and several organs including pleura, pericardium, lungs, joints, and striated muscle including diaphragm and myocardium [102]. Though the overall risk in the absence of risk factors is very low, its incidence appears to be between 1 and 4% when these are present [8]. Thus, administration of gadolinium in patients with these conditions must be made if expected benefits outweigh risks, using a macrocyclic chelate in the lowest possible dose and avoiding repeat exposure. Post-procedure haemodialysis of all patients with end-stage renal failure should be considered.

### Primary hyperaldosteronism (Conn’s syndrome)

This is a much more common cause of hypertension than previously thought and is present in up to 20% of patients with resistant hypertension [103]. Given the high incidence of adrenal incidentalomata, direct imaging is usually not recommended. Instead, initial biochemical assessment followed by imaging (which raises pre-test likelihood and therefore post-test likelihood ratios in the event of a positive test) is recommended in hypertensive patients with resistant hypertension, hypokalemia or features of secondary hypertension. The most frequent cause is adrenal adenoma, and makes up 65% of cases. Bilateral adrenal hyperplasia is responsible for 30% of cases and the remainders are due to adrenal carcinoma or extra-adrenal tumours. While screening is based on high plasma aldosterone and high plasma aldosterone/plasma renin activity ratio and confirmation is done by demonstrating a high 24-hour urinary aldosterone in the course of a high dietary sodium intake, MR is indicated for localization of the causative lesion. Most adenomas are iso- or hypo-intense relative to the liver on T1-weighted images and slightly hyperintense on T2-weighted images, while with chemical shift imaging the signal characteristically decreases on the out-of-phase images. MR has very high specificity for detecting aldosterone-producing adenomata and sensitivity similar to that of CT imaging [104].

### Cushing’s syndrome

**Figure 9:**
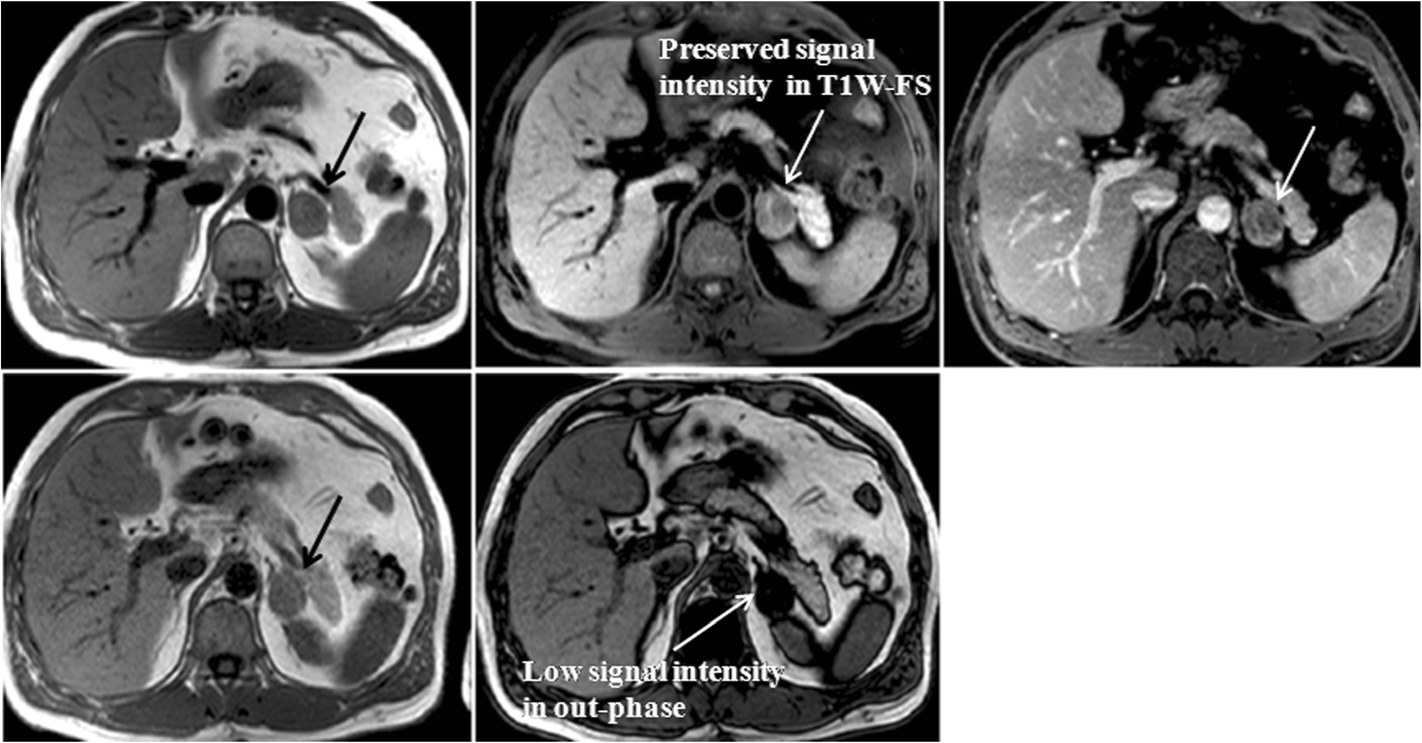
**MR study showing an adrenal adenoma.** The upper row shows, from left to right, T1W, T1W-FS and VIBE sequences. The lower row shows, from left to right, in-phase and out-phase sequences. An adrenal adenoma is visible on the left adrenal gland, with preserved signal intensity in the T1W-FS sequence and low signal intensity in the out-phase sequence.

The exact prevalence of Cushing’s syndrome amongst patients with resistant hypertension is uncertain, but it is estimated that approximately 3 to 6% of cases of secondary hypertension are due to corticosteroid overproduction [105]. Hypertension in Cushing’s syndrome is significantly correlated with the duration of hypercortisolism and results from the interplay between several pathophysiological mechanisms regulating plasma volume, peripheral vascular resistance and cardiac output. Detection is important for therapeutic and also for prognostic reasons, as target organ damage in this condition is more severe than in primary hypertension [106] and the overall cardiovascular risk is increased because the disorder is associated with other concomitant major risk factors such as diabetes mellitus, metabolic syndrome and dyslipidemia. Cushing’s syndrome may be due to steroid use, ectopic ACTH, pituitary microadenoma and adrenal adenoma. Initial screening is carried out with an overnight 1 mg dexamethasone suppression test, which is preferred over measurement of 24-hour urine free cortisol. In order to differentiate pituitary dependent syndromes from non-pituitary causes, combination of a CRH stimulation test with a dexamethasone suppression test and measurement of ACTH can be done. Finally, localization, needed before surgical therapy, is done by MR or CT [107]. If test results indicate an ACTH-independent lesion, abdominal CT or MR scanning are indicated. Figure 9 shows an MR study of an adrenal adenoma visible on the left adrenal gland, including T1W, T1W-FS and VIBE sequences (top), and in-phase and out-phase sequences (bottom). If the process is ACTH dependent, a high-dose dexamethasone suppression test combined with cranial MR may aid in localizing the lesion [108]. Hypertension in Cushing’s syndrome usually resolves with surgical removal of the tumour, but some patients require pharmacological anti-hypertensive treatment both pre- and post-operatively.

### Phaeochromocytoma

This is an unusual but prognostically important secondary cause of hypertension. Again, the prevalence of this condition in resistant hypertension is unknown but it is considered to be up to 0.6% in the general hypertensive population [109]. Phaeochromocytoma is characterized by increased blood pressure and blood pressure variability [110] which constitutes an additional independent risk factor for cardiovascular morbidity and mortality. Thus, detection is important for therapeutic and prognostic reasons. Diagnosis is based on measurement of plasma free metanephrines and of 24-hour urinary catecholamine levels. Imaging techniques are indicated for localizing and staging the tumour, but not for tissue characterization as this tumour can undergo a number of forms of pathologic degeneration affecting its imaging features. Thus, though 65% of phaeochromocytomas are characteristically solid, hypervascular masses with low signal intensity on T1-weighted, high signal intensity on T2-weighted MR images and enhancement after gadolinium, a wide range of imaging appearances can be found, mimicking other lesions both benign and malignant. For instance, phaeochromocytomas may be dark on T2-weighted images, or they may include haemorrhagic, cystic, fibrotic and calcific changes, or they may have so much fat as to be misdiagnosed as an adenoma. Results on CT and radionuclide imaging can also be inconsistent.

### Aortic coarctation

This congenital defect is present in 0.2% of hypertensive adults [111]. Coarctation of the aorta imposes significant afterload on the left ventricle (LV), which results in increased wall stress and compensatory ventricular hypertrophy. The mechanism by which hypertension appears in these patients is not completely understood. Several mechanisms have been postulated: mainly the mechanical obstruction hypothesis, the renin-angiotensin–mediated humoral hypothesis or Goldblatt-type phenomenon and the neural theory. According to the mechanical obstruction theory, a higher blood pressure is required to maintain flow through the coarcted segment and collateral vessels. As a result, the stroke volume ejected into the limited aortic receptacle produces a higher pressure proximal to the coarctation. This theory, though, fails to explain features such as the abnormal blood pressure response to exercise after repair, the development of early hypertension in spite of early effective repair and the development of end-organ damage late after repair [112]. The humoral theory postulates activation of the renin-angiotensin system secondary to reduction of renal blood flow but plasma renin activity has not been consistently found to be elevated in these subjects and, while it is reasonable to hypothesize that an increase in the renine angiotensin system contributes to hypertension in patients with coarctation, its role in the development of hypertension at rest and during exercise after a successful repair is still unclear [113]. Some studies have suggested reduced baroreflex sensitivity as a cause of abnormal blood pressure regulation after coarctation repair [114], while the concomitant effect of other associated cardiac anomalies and neurohumoral changes that occur in heart failure cannot be discarded.

**Figure 10:**
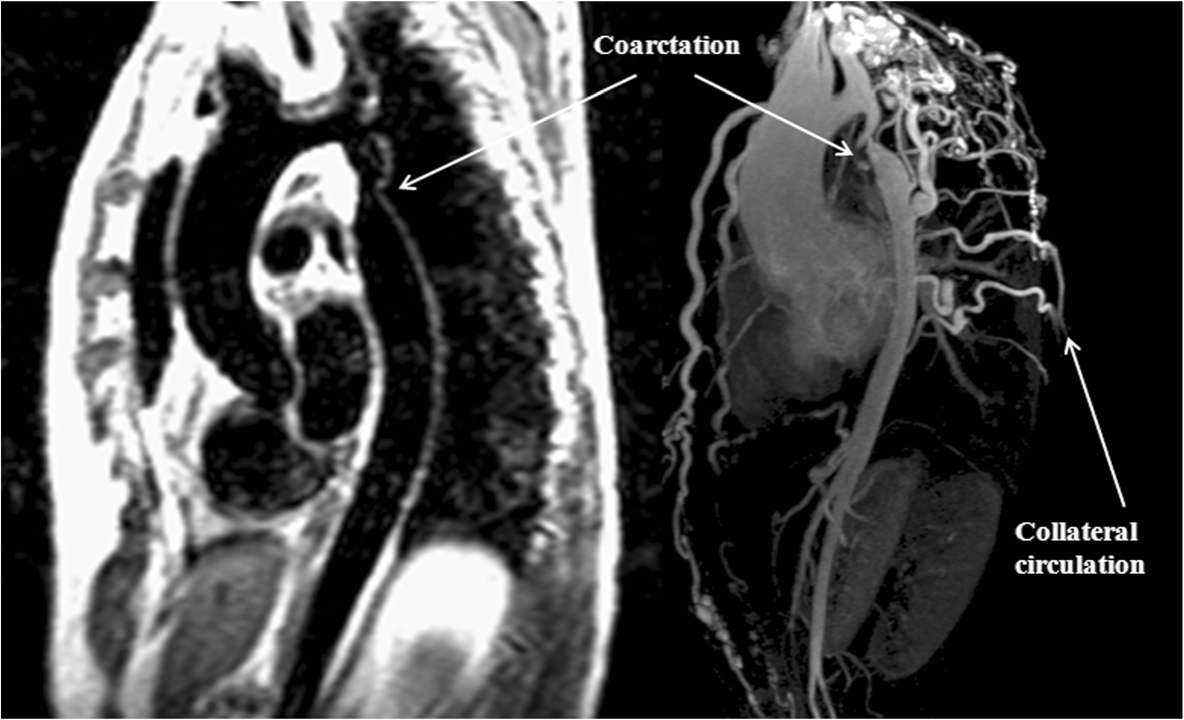
**T1W-TSE (left) and 3D-MRA (right) of two patients with aortic coarctation.** In both cases coarctation of the aorta is seen in a juxtaductal position, with significant collateral circulation seen in the 3D-MRA sequence.

Echocardiography, both transthoracic and transoesophageal, is usually the imaging technique of choice for initial assessment [115], but for further evaluation contrast angiography, cardiovascular computed tomography and CMR have been used [116]. Even though contrast angiography is still considered the gold standard for evaluation of aortic coarctation, the use of CMR has increased over the past few years. The availability of different techniques such as cine sequences, phase contrast imaging and 3D angiography allow for the accurate and reproducible description of aortic anatomy, presence of collaterals and quantification of severity through peak velocity across the stenosis and amount of collateral flow, which help in decision making [117]. The ability to perform serial follow up studies both before and after repair with no ionising radiation is a distinct advantage for CMR over CT particularly in this younger cohort of patients. Figure 10 shows T1W-TSE and 3D-MRA sequences of two patients with juxtaductal aortic coarctation and significant collateral circulation.

Aortic coarctation is associated with functional aorto-ventricular disturbances, such as abnormal myocardial perfusion reserve with sub-endocardial ischaemia, and increased central aortic stiffness that leads to ascending aorta dilatation and increased LV mass. These conditions persist after surgery and may contribute to increase cardiovascular mortality and morbidity. Finally, re-coarctation of the aorta may lead to worsening systemic hypertension, coronary artery disease and/or congestive cardiac failure. CMR follow-up after coarctation repair will detect re-coarctation as well as those with aortoventricular disturbances, who require more frequent non-invasive surveillance [118].

### Future directions

The unique potential of CMR in hypertensive heart disease implies a high number of indications to be developed in the future, both in diagnosis and in risk stratification of future cardiovascular events. These include analysis and quantification of regional myocardial mechanics, which will allow detection of subtle decay in systolic function, detection and quantification of diffuse myocardial fibrosis, assessment of atrial mechanics, and assessment and quantification of aortic flow mechanics.

The prognostic value of measuring diffuse and focal myocardial fibrosis in predicting future cardiovascular events such as heart failure or arrhythmias also hold promise with a potential role for monitoring the response to antihypertensive, antifibrotic drugs. Parameters of atrial function and wall fibrosis should also be tested for prediction of developing atrial fibrillation.

## Conclusions

CMR provides a comprehensive assessment of hypertensive cardiovascular disease. In less than an hour, ventricular and aortic measurements are obtained with high accuracy and reproducibility, the pathophysiological consequences of hypertension can be assessed and several quantitative parameters can be obtained with high reproducibility and accuracy that can be of use in the follow up of these patients. Concurrently, several secondary causes of hypertension can be ruled out in the same study. With respect to prognostic assessment, the development of T1 mapping sequences for quantification of myocardial fibrosis might play an important role in the risk stratification of hypertensive heart disease.

## Competing interests

The authors declare that they have no competing interests.

## Authors’ contributions

AMMG carried out the review of literature, manuscript design and drafting. RHM has been involved in revising the manuscript critically for important intellectual content, carried out the final revision and approved the manuscript to be submitted. Both authors have read and approved the final manuscript.
